# Associations Between Generative AI Use and Facial Expression-Derived Central Executive Network Indices: A Pilot Study

**DOI:** 10.3390/brainsci16010058

**Published:** 2025-12-30

**Authors:** Keisuke Kokubun, Yoshinori Yamakawa, Anna Yoshida, Shinichiro Sanji

**Affiliations:** 1 Graduate School of Management, Kyoto University, Kyoto 606-8501, Japan; 2 PwC Consulting LLC, Tokyo 100-0004, Japan

**Keywords:** central executive network, default mode network, generative AI, gray matter volume

## Abstract

**Background/Objectives:** The rapid diffusion of generative AI has raised concerns about its potential influence on human cognition, particularly during creative work. This pilot study explored task-related associations between generative AI use and facial expression-derived indices that have previously been shown to correlate with gray matter volume in the default mode network (DMN) and central executive network (CEN). **Methods:** Thirty-three business professionals completed three AI-supported writing tasks involving concept generation, concept combination, and a mixed task. **Results:** The results showed a statistically robust reduction in the CEN-related facial expression index during the concept combination task, whereas no corrected changes were observed during concept generation or the mixed task. In addition, higher creative self-efficacy was associated with smaller reductions in the CEN-related index. **Conclusions:** Given the indirect nature of the facial expression measures, the absence of a control condition, and the exploratory design, these findings should be interpreted cautiously and primarily as hypothesis-generating. Future research using controlled designs and direct neuroimaging methods is needed to clarify the cognitive and neural mechanisms underlying AI-assisted creativity.

## 1. Introduction

The advent of generative artificial intelligence (AI) has substantially transformed contemporary work practices. Users of generative AI tools are reported to expend less effort on tasks such as information searching and curation, instead reallocating cognitive resources toward verifying and refining AI-generated outputs [1]. In particular, large language models (LLMs)—a class of generative AI systems specialized in natural language processing—streamline information access by reducing the need to consult and integrate multiple external sources. Empirical studies indicate that LLM use can reduce various forms of cognitive load and facilitate comprehension and information retrieval compared with traditional web-based search methods [2]. Related work further reports reductions in frustration and perceived effort during information search, accompanied by gains in productivity and sustained task engagement [3].

Despite these efficiency gains, concerns have emerged regarding the implications of generative AI for learning, creativity, and writing-related cognition. By generating content on demand from minimal prompts, LLMs can provide rapid drafts that may support ideation and time efficiency. However, several studies suggest that such support does not necessarily translate into improved knowledge acquisition or transfer. For example, while AI-assisted writing has been shown to improve short-term task performance, such as essay scores, it does not reliably enhance conceptual understanding or long-term learning outcomes [4]. Moreover, AI tools that minimize the need for reflection, revision, or source integration may allow users to bypass cognitively demanding processes essential for deep learning and schema construction [2,4]. Experimental evidence further indicates that reliance on generative AI can reduce creative writing performance relative to unaided or traditionally supported writing tasks [5].

The ease of access to AI-generated answers may also encourage passive information consumption. Such patterns of use have been linked to superficial engagement, weakened critical thinking, reduced depth of comprehension, and the impaired formation of durable memory representations [6]. When problem solutions are immediately available, users may be less inclined to engage in independent problem-solving or exploratory reasoning [7]. From a cognitive perspective, insufficient stimulation and sustained reliance on external tools may undermine processes associated with cognitive development and memory consolidation [7]. These concerns are closely related to the broader concept of cognitive offloading, whereby individuals delegate internal cognitive processes to external aids, potentially altering patterns of cognitive engagement over time [8,9]. Consistent with this view, multiple studies report that excessive reliance on generative AI is associated with reduced critical thinking and increased dependency on automated systems [1,10,11,12,13].

Individual differences appear to play a central role in shaping how generative AI is used and how it affects cognitive engagement. Self-efficacy—defined as confidence in one’s ability to plan, organize, and execute tasks—has been identified as an important moderator of AI use [12,14]. Users with lower self-efficacy tend to rely more heavily on AI-generated outputs, often prioritizing immediate solutions over effortful cognitive processing [15,16,17]. By contrast, individuals with greater domain knowledge and higher cognitive ability are more likely to use digital tools strategically, actively evaluating, integrating, and transforming information rather than passively accepting it [18,19]. Recent comparative studies further suggest that high-ability learners tend to use LLMs as supports for active learning, whereas low-ability learners are more likely to substitute AI outputs for their own cognitive effort, potentially reducing productive cognitive load [20].

Insights from neuroscience provide an additional perspective on how different forms of information processing engage the brain. Functional magnetic resonance imaging (fMRI) studies have shown that active web searching engages broad neural networks associated with decision-making, working memory, and executive control, including the dorsolateral prefrontal cortex, anterior cingulate cortex (ACC), and hippocampus [21,22]. Such engagement reflects the cognitively demanding nature of searching, which requires goal maintenance, semantic integration, and strategic evaluation. Information-seeking behavior is also supported by neural systems involved in reward prediction and uncertainty resolution, with dopaminergic pathways encoding the anticipated value of information and sustaining motivation during exploratory behavior [19].

In contrast, emerging evidence suggests that reliance on generative AI may be associated with reduced engagement of cognitive and neural processes during writing tasks. For example, experimental work using electroencephalography (EEG) has shown that participants using generative AI during essay writing exhibited lower integrative neural activity compared with those using traditional search tools or no external assistance [20]. These patterns were accompanied by behavioral indicators of reduced initiative and engagement, such as passive acceptance of AI-generated text and limited revision. Content analyses further indicated reduced originality and increased repetitiveness in AI-assisted outputs [20]. While such findings do not establish causal effects on brain function, they raise important questions about how external cognitive support may alter patterns of executive engagement.

From a network neuroscience perspective, creative cognition has been linked to interactions between the default mode network (DMN) and the central executive network (CEN). The CEN—encompassing regions such as the dorsolateral prefrontal cortex, dorsal ACC, and lateral parietal cortex—is associated with sustained attention, working memory, and cognitive control, whereas the DMN supports internally oriented thought, spontaneous association, and idea generation [23,24,25]. Although these networks are typically anticorrelated at rest, converging evidence suggests that they may cooperate during creative tasks [23,24,25,26,27,28,29]. Lesion and network-control studies further indicate a functional dissociation between creative processes: concept generation appears to rely more heavily on DMN-related regions, whereas concept combination and evaluation depend on CEN-related control mechanisms [30,31,32].

Against this background, the present pilot study examined task-specific associations between generative-AI–supported creative writing and facial expression-derived indices that have previously been shown to correlate with gray matter volume in the DMN and CEN. Participants completed three AI-supported tasks designed to emphasize concept generation, concept combination, and a mixed process. By focusing on changes in facial expression-derived indices across tasks, and by examining their relationships with self-efficacy and self-evaluations of performance, this study aims to provide exploratory evidence regarding how different forms of AI-assisted creative work are associated with executive- and default-mode–network–related correlates. Given the indirect nature of the measures and the absence of a control condition, the findings are intended to be hypothesis-generating rather than conclusive.

The hypotheses of this study are as follows:

**H1.**  *Concept generation (Task 1) will reduce facial expression-derived indices associated with the DMN.*

**H2.**  *Concept combination (Task 2) will reduce facial expression-derived indices associated with the CEN.*

**H3.**  *Self-efficacy will be associated with changes in DMN-related indices during concept generation (Task 1).*

**H4.**  *Self-efficacy will be associated with changes in CEN-related indices during concept combination (Task 2).*

**H5.**  *Self-evaluation will be associated with changes in DMN-related indices during concept generation (Task 1).*

**H6.**  *Self-evaluation will be associated with changes in CEN-related indices during concept combination (Task 2).*

## 2. Materials and Methods

### 2.1. Participants

A priori power analysis for paired comparisons was conducted using G*Power software (version 3.1.9.7, Heinrich Heine University Düsseldorf, Düsseldorf, Germany). Assuming a two-sided significance level of 10%, statistical power of 80%, and a moderate effect size (Cohen’s d = 0.5) [33], the required sample size was estimated to be 27 participants. To account for potential dropouts, 33 individuals (21 men, 12 women) were recruited and participated in the study between 5 and 7 August 2025. The mean age was 32.5 ± 6.9 years.

Participants were randomly selected from employees of a technology consulting firm (PwC). All participants reported no history of neurological, psychiatric, or other medical conditions affecting the central nervous system. Written informed consent was obtained from all participants prior to participation, and anonymity was ensured. All procedures were conducted in accordance with relevant guidelines and regulations. The study was approved by the Ethics Committee of the Tokyo Institute of Science (Approval Number 2023137).

### 2.2. Task

First, participants received a brief lecture from the instructor on how to use the Quick-BHQ and then completed a questionnaire. Subsequently, they engaged in three sessions, each consisting of facial expression measurement using the Quick-BHQ (quantification of facial expressions linked to brain structure as explained later) and a task. Each session lasted seven minutes, and the task content differed across the three sessions.

In Test 1, participants were given the following introductory instruction:

*“Let us explore new business ideas leveraging brain health, using generative AI (Chat PwC). Please write down your top three ideas from the ones generated. The format of your response is free.”*

Along with this instruction, three example responses were provided:

1. Interactive Cognitive Gaming Platform: Interactive games represent an innovative form of cognitive training. Through gameplay that leverages users’ knowledge and provides real-time feedback, they can promote brain health in an enjoyable way.
2. Mental Resilience Enhancement Plan: This plan offers a novel form of education and experiential learning that simultaneously strengthens stress management and problem-solving abilities. Given the growing demand for mental toughness in modern society, a program centered on this theme offers clear originality.
3. Relaxation Retreat: A relaxation retreat emphasizes comfort and peace, offering a new form of experiential program. It represents an innovative approach that provides specialized retreats designed to heal the mind and brain through coexistence with nature.

In Test 2, participants were given the following instruction:

*“From the ideas you listed earlier, please select one. Using generative AI (Chat PwC), examine its business potential and summarize the result in approximately 300 Japanese characters.”*

Chat PwC refers to an internally deployed enterprise conversational AI environment at PwC. Because this system is continuously updated and its internal model specifications are not publicly disclosed, it does not have a fixed or publicly identifiable software version number.

As an example, if the participant selected the idea “Interactive Cognitive Gaming Platform,” the following sample response was provided:

*“The Interactive Cognitive Gaming Platform addresses a promising demand in the educational gaming market, which is expected to grow by 15% annually. The overall market size is approximately 200 billion USD, and we aim to capture a 0.1% to 0.5% share. The revenue model adopts a monthly subscription, with projected first-year revenue ranging from 500,000 to 2 million USD and an anticipated profit margin of about 15%. Furthermore, by targeting over 70% user satisfaction and more than 50% monthly retention, the platform seeks to enhance customer experience and build long-term user loyalty.”*

(289 characters)

In Test 3, participants were given the following instruction:

*“Based on the idea and its business potential you considered earlier, please use generative AI (Chat PwC) to create a catchphrase for the idea and a key message (within 100 Japanese characters) that explains it.”*

As an example, the following sample response was provided:

- Title: Brain Adventure: A Health Revolution through Interactive Gaming
- Key Message: “Brain Adventure” is a platform that enhances cognitive abilities while having fun with interactive games. Targeting a share in the rapidly growing educational gaming market, it ensures profitability through a subscription model and aims for high user satisfaction and retention. (130 characters)

### 2.3. Psychological Measures

Prior to the experimental tasks, participants completed a measure of creative self-efficacy adapted from a previously validated scale [34]. The scale consisted of three items assessing participants’ confidence in their ability to engage in creative problem solving when using AI assistance (e.g., “I have confidence in my ability to solve problems creatively when working with AI assistance”). Responses were recorded on seven-point Likert scales ranging from 1 (strongly disagree) to 7 (strongly agree), with higher scores indicating greater creative self-efficacy.

After completing all tasks, participants were asked to provide self-evaluations of their outputs for each task. Responses were recorded on five-point Likert scales ranging from 1 (not at all accomplished) to 5 (very well accomplished).

### 2.4. Facial Expression Information

**Figure 1:**
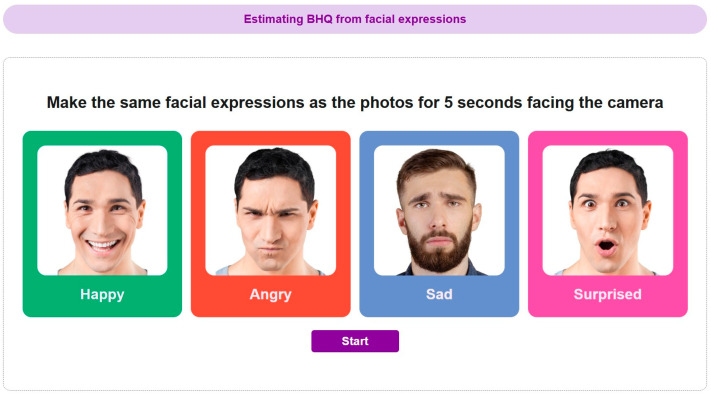
Facial expressions imitated by participants during data collection. These expressions were used as inputs for calculating facial expression-derived indices previously shown to correlate with brain structural measures; they do not represent direct measurements of neural activity.

Facial expressions were measured using a proprietary application developed by Panasonic Corporation [35,36]. During the assessment, participants were seated in front of a camera and instructed to imitate facial expressions displayed as photographs on a computer screen. As shown in Figure 1, four basic facial expressions—happiness, anger, sadness, and surprise—were assessed. The photographs were presented in a randomized order for each participant, and facial expression data were recorded while participants imitated each expression for 5 s. These facial expression data were transformed into indices derived from a previously validated algorithm that has been shown to correlate with gray matter volume (GMV) measures of the DMN and CEN obtained from MRI in independent samples [36]. Importantly, these indices do not represent direct measures of brain activity or structure, but rather indirect facial expression–based proxies whose interpretation should be treated with caution.

### 2.5. Data Analysis

From the facial expression information collected, facial expression-derived indices previously shown to correlate with GMV of the CEN and DMN were calculated. We also reported, for reference, results related to facial expressions reflecting whole-brain GMV, although these were not the primary focus of the statistical analyses. For these values, the incremental changes (Δ) across stages were calculated, and independent *t*-tests were conducted to examine whether the increments significantly differed from zero. Considering that this is a pilot study, the criterion for significance was set at 10% on both sides, and correction was performed for multiple comparisons using the Bonferroni test. This relatively liberal threshold was adopted to reduce the risk of Type II error (i.e., failing to detect potentially meaningful effects) in an exploratory study with a limited sample size. Importantly, results meeting this exploratory threshold are clearly distinguished from those surviving multiple-comparison correction, which are interpreted as statistically robust. In addition, effect sizes (Cohen’s d) and 90% confidence intervals (90% CIs) were reported where applicable to facilitate interpretation of the magnitude and precision of the observed effects. All statistical analyses were performed using IBM SPSS Statistics version 28 (IBM Corp., Armonk, NY, USA).

## 3. Results

Table 1 presents the results of the Δ values and *t*-tests. From the first to the second task, Δ whole-brain 1 (mean = 1.140, *p* = 0.061) was significantly greater than zero at the 10% level. Given the exploratory nature of this pilot study, a 10% significance threshold was adopted to reduce Type II error, while all results surviving multiple-comparison correction are explicitly indicated. From the second to the third task, Δ DMN 2 (mean = −0.790, *p* = 0.093) and Δ CEN 2 (mean = −1.560, *p* = 0.032) were significantly less than zero at the 10% level. Among these, only Δ CEN 2 satisfied the Bonferroni-adjusted significance threshold. The magnitude of this effect was small to moderate (Cohen’s d = −0.39), and the corresponding 90% confidence interval excluded zero, indicating a directional reduction in the CEN-related facial expression-derived index during the concept combination task under the exploratory criterion.

**Figure 2:**
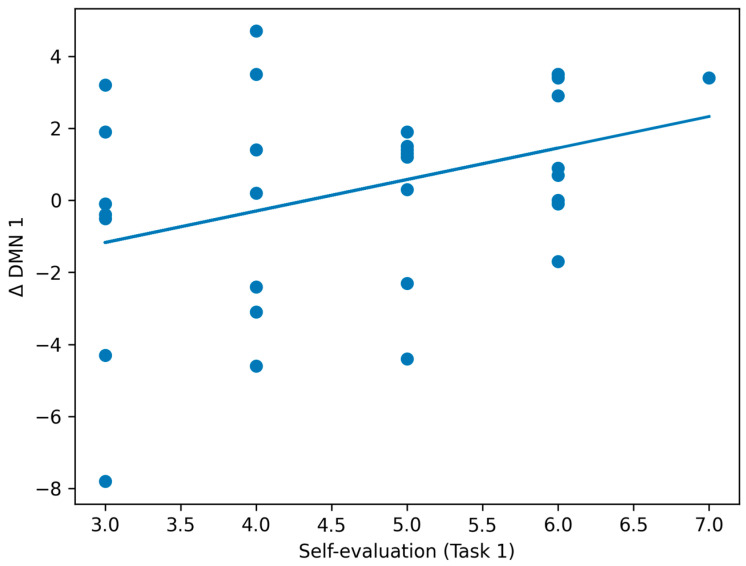
Association between self-evaluation of Task 1 and changes in the facial expression-derived DMN index (Δ DMN 1). The solid line represents the linear regression, and dots indicate individual participants. Although the association reached statistical significance (r = 0.36, *p* = 0.038), the effect size was small (r^2^ = 0.13), indicating substantial overlap among individuals. The DMN index represents an indirect facial expression–based proxy previously shown to correlate with MRI-based structural measures and does not reflect direct neural activity.

**Figure 3:**
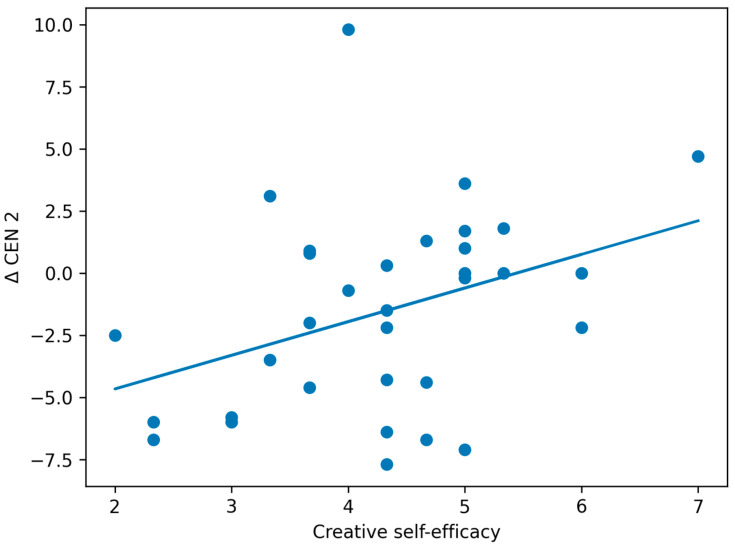
Association between creative self-efficacy and changes in the facial expression-derived CEN index during the concept combination task (Δ CEN 2). The solid line represents the linear regression, and dots indicate individual participants. A statistically significant association was observed (r = 0.37, *p* = 0.033); however, the effect size was small (r^2^ = 0.14), suggesting a limited behavioral association despite statistical significance. Values represent indirect facial expression-derived indices and do not correspond to direct measures of neural activity.

Next, we conducted a correlation analysis among creative self-efficacy, self-evaluations for each task, and the Δ values. The results showed significant correlations between creative self-efficacy and Δ CEN 2 (*r* = 0.372, *p* = 0.033), as well as between the first self-evaluation and Δ DMN 1 (*r* = 0.363, *p* = 0.038). Both correlations remained significant after Bonferroni correction, meeting the significance threshold of *p* < 0.05. These relationships are illustrated in scatterplots in Figure 2 and Figure 3. In addition, a significant correlation was observed between the second self-evaluation and Δ whole-brain 2 (*r* = 0.394, *p* = 0.023).

Taken together, these results support hypotheses H2, H4, and H5, while H1, H3, and H6 were rejected.

## 4. Discussion

In this pilot study, we examined task-specific associations between generative-AI–supported creative writing and facial expression-derived indices previously shown to correlate with gray matter volume in the default mode network (DMN) and central executive network (CEN). Among the three tasks examined, the most robust finding was a reduction in the CEN-related index during the concept combination task. This effect survived correction for multiple comparisons, whereas no corrected changes were observed during the concept generation or mixed tasks.

Importantly, these findings should not be interpreted as evidence of changes in neural activity or brain structure. The indices used in this study are indirect facial expression–based proxies and do not provide direct measures of brain function. Accordingly, the present results are best interpreted as associations between task characteristics and facial expression patterns linked to executive-network–related structural correlates.

Furthermore, the statistically significant correlations observed in this study were characterized by small r-squared values (approximately 13–14%), indicating substantial overlap among individuals. From a behavioral perspective, these effect sizes suggest weak associations rather than strong or practically decisive effects. Accordingly, the present findings should be interpreted as having limited behavioral relevance, consistent with the exploratory and pilot nature of the study.

Previous neuroimaging research has emphasized the role of the CEN in the controlled integration and evaluation of ideas during creative cognition [30,31]. Within this broader literature, the observed reduction in the CEN-related facial expression-derived index during the concept combination task may be interpreted as consistent with the possibility that external cognitive support alters the engagement of executive control processes. However, the present study does not permit inferences regarding functional substitution, reductions in cognitive load, or diminished neural engagement, and any such interpretations should therefore be regarded as speculative.

Although several theoretical and empirical models have proposed cooperative dynamics between the DMN and the CEN during creative tasks [23,24,25,26], the current data do not provide empirical support for such mechanisms. While a non-significant trend toward reduced DMN-related indices was observed during the concept combination task, this effect did not survive correction for multiple comparisons and should not be overinterpreted. Accordingly, references to DMN–CEN cooperation are included here solely as conceptual background rather than as conclusions supported by the present findings.

Individual differences further qualified the observed associations. Participants with higher creative self-efficacy exhibited smaller reductions in the CEN-related index during the concept combination task, suggesting that user characteristics may moderate how individuals engage with external cognitive tools, including generative AI [12,17]. Similarly, higher self-evaluation during the concept generation task was associated with changes in DMN-related indices; however, these associations should be interpreted cautiously given the exploratory nature of the analyses.

The absence of significant changes during the mixed task may reflect competing task demands or limitations in the sensitivity of facial expression-derived indices to capture more complex or overlapping cognitive processes. Taken together, the findings suggest that associations between generative AI use and executive-related facial expression indices are task-dependent and moderated by individual characteristics. Given the exploratory design, the lack of a control condition, and the indirect nature of the measurement approach, the present results should be regarded as hypothesis-generating and as a foundation for future research employing controlled experimental designs and direct neuroimaging methods.

## 5. Limitations

This study has several limitations. First, this study showed that AI-related tasks caused changes in facial expressions related to CEN, but it did not show that CEN itself changed. Second, this study did not include a control group, so we cannot exclude the possibility that factors other than the intervention may have influenced the results. Third, because this study was conducted on Japanese participants, caution is required regarding whether the results can be applied to other countries. Fourth, the small sample size limits the generalizability of the results. Fifth, background information not included in this study, such as socioeconomic status such as income and differences in the communities to which participants belong, may have influenced the results.

## 6. Conclusions

This pilot study examined task-specific associations between generative AI use and facial expression-derived indices previously shown to correlate with executive- and default-mode–network–related structural characteristics. The findings indicate that, among the tasks examined, only generative-AI–supported concept combination was associated with a robust reduction in the CEN-related facial expression index, whereas concept generation and mixed tasks showed no corrected changes. Importantly, these results do not demonstrate changes in neural activity or brain structure but instead highlight associations observable at the level of indirect facial expression proxies. The moderating role of creative self-efficacy further suggests that individual differences may shape how users cognitively engage with generative AI tools. Given the exploratory nature of the study, the lack of a control condition, and the limited sample size, the present findings should be interpreted with caution. Nevertheless, they provide a basis for future hypothesis-driven research employing controlled experimental designs and direct neuroimaging approaches to better understand the cognitive implications of AI-assisted creative work.

## Figures and Tables

**Table 1:** Changes in facial expression-derived indices across tasks.

	Mean	SD	t	*p*	Cohen’s d	90% CI (Mean)
Changes after Task 1						
Δ whole-brain 1	1.14	3.37	1.942	0.061 *	0.34	[0.146, 2.133]
Δ DMN 1	0.31	2.854	0.628	0.534	0.11	[−0.529, 1.154]
Δ CEN 1	0.85	4.208	1.162	0.254	0.2	[−0.389, 2.092]
Changes after Task 2						
Δ whole-brain 2	−0.580	3.814	−0.881	0.385	−0.15	[−1.709, 0.540]
Δ DMN 2	−0.790	2.636	−1.730	0.093 *	−0.30	[−1.571, −0.017]
Δ CEN 2	−1.560	3.995	−2.244	0.032 *^,†^	−0.39	[−2.739, −0.383]
Changes after Task 3						
Δ whole-brain 3	−0.420	3.24	−0.736	0.467	−0.13	[−1.371, 0.540]
Δ DMN 3	−0.040	3.215	−0.076	0.94	−0.01	[−0.990, 0.905]
Δ CEN 3	0.33	3.885	0.484	0.632	0.09	[−0.818, 1.473]

Note: * *p* < 0.10 indicates exploratory significance. ^†^ *p* < 0.05 indicates effects surviving Bonferroni multiple-comparison correction. Cohen’s d represents standardized mean differences from zero (one-sample effect size). We report 90% confidence intervals consistent with the two-sided α = 0.10 used in this pilot study. All values are facial expression-derived indices previously shown to correlate with gray matter volume in the DMN and CEN and do not represent direct neural measures.

## Data Availability

The data presented in this study are available on request from the corresponding author due to the need to protect the privacy of participants.
